# Prognostic factors for overall survival and safety of trans-arterial chemoembolization (TACE) with irinotecan-loaded drug-eluting beads (DEBIRI) in patients with colorectal liver metastases

**DOI:** 10.2478/raon-2024-0023

**Published:** 2024-03-30

**Authors:** Maja Sljivic, Masa Sever, Janja Ocvirk, Tanja Mesti, Erik Brecelj, Peter Popovic

**Affiliations:** Faculty of Medicine Ljubljana, Ljubljana, Slovenia; Clinical Institute of Radiology, University Medical Centre Ljubljana, Ljubljana, Slovenia; Faculty of Medicine Belgrade, Serbia; Institute of Oncology, Ljubljana, Slovenia; University of Primorska, Faculty of Health Sciences, Isola, Slovenia

**Keywords:** colorectal cancer, liver metastases, irinotecan, drug-eluting beads, transarterial chemoembolization, survival

## Abstract

**Background:**

Transarterial chemoembolisation with irinotecan-loaded drug-eluting beads (DEBIRI TACE) can be considered in patients with unresectable colorectal cancer liver metastases (CRLM) who progress after all approved standard therapies or in patients unsuitable for systemic therapy.

**Patients and methods:**

Between September 2010 and March 2020, thirty patients (22 men and 8 women; mean age 66.8 ± 13.2) were included in this retrospective study. DEBIRI TACE was conducted in 43% of patients unsuitable for systemic therapy as a first-line treatment and 57% as salvage therapy after the progression of systemic therapy. All the patients had liver-limited disease. In the case of unilobar disease, two treatments were performed at four-week intervals, and in the case of bilobar disease, four treatments were performed at two-week intervals. All patients were premedicated and monitored after the procedure. Adverse events were graded according to the Cardiovascular and Interventional Radiological Society of Europe (CIRSE) classification system for complications.

**Results:**

The median overall survival (OS) from the beginning of DEBIRI TACE in the salvage group was 17.4 months; in the group without prior systemic therapy, it was 21.6 months. The median overall survival of all patients was 17.4 months (95% confidence interval [CI]: 10.0–24.7 months), and progression-free survival (PFS) was 4.2 months (95% CI: 0.9–7.4 months). The one-year survival rate after the procedure was 61%, and the two-year rate was 25%. Univariate analysis showed better survival of patients with four or fewer liver metastases (*p = 0.002*). There were no treatment-related deaths or grade 4 and 5 adverse events. Nonserious adverse events (Grades 1 and 2) were present in 53% of patients, and Grade 3 adverse events were present in 6% of the patients.

**Conclusions:**

DEBIRI TACE is a well-tolerated treatment option for patients with liver metastases of colorectal cancer. Patients with four or fewer liver metastases correlated with better survival.

## Introduction

Colorectal cancer (CRC) is Europe’s second most frequently diagnosed malignancy and the second most common cause of death due to cancer (excluding skin carcinomas).^1,2^ At the time of diagnosis, 25% of patients have already developed CRC metastases, and 25−35% of patients will develop metastases in the later stages of their disease.^3^ The liver is the most common site of CRC metastases (CRLM). The disease progression in the liver is a significant source of complications and death.^4,5^ The only curative treatment option for CRLM is surgical resection.^4,6^ Unfortunately, most (approx. 70−80%) metastases are unresectable.^7,8^ The therapy of choice for non-resectable CRLM is multiagent systemic chemotherapy, such as FOLFOX or FOLFIRI, and targeted agents, including epidermal growth factor receptor (EGFR) and vascular endothelial growth factor (VEGF) inhibitor.^7,8^ The median overall survival of first-line chemotherapy ranges from 12 to 23 months, which is further increased by another two months with the addition of anti-VEGF agent bevacizumab. Median overall survival (OS) reaches around 30 months with a multi-line treatment plan.^7,8^

Conventional transarterial chemoembolisation (TACE) is a selective intraarterial administration of chemotherapeutic agents in combination with Lipiodol. The newer method uses drug-eluting beads (DEB) that cause embolisation and release chemotherapeutic agents into the targeted tissue. The most used chemotherapeutic agent in TACE for CRLM is irinotecan (transarterial chemoembolisation with irinotecan-loaded drug-eluting beads, DEBIRI TACE).^9^ Based on current European Society for Medical Oncology (ESMO) guidelines, TACE should be considered a possible treatment option when patients with metastatic liver-limited disease do not respond to systemic chemotherapy.^7,8^ Studies have shown that DEBIRI TACE is an effective and safe procedure, with serious high-grade adverse reaching up to 11%.^9,10,11,12,13,14^ On the other hand, getting as much data as possible on how DEBIRI TACE works in real life and finding a group of patients who would benefit the most from this type of treatment is essential. Studies examining prognostic factors for determining survival and treatment efficacy in patients with CRLM treated with DEBIRI TACE are rare.^11,12,13^ Research is ongoing, and most authors suggest that further research is needed. Therefore, additional clinical and radiological prognostic factors are required to choose the appropriate patient profile for treatment with DEBIRI TACE.

This retrospective study investigated the safety and prognostic factors in predicting overall survival in patients with CRLM treated with DEBIRI TACE.

## Patients and methods

### Study design and patient selection

This single-centre retrospective study was approved by the Republic of Slovenia National Medical Ethics Committee (0120-115/2020/9). The study complied with the protocol and principles in the Declaration of Helsinki. Between September 2010 and March 2020, 30 patients with unresectable liver metastases of colorectal cancer who did not respond to systemic therapy, had contraindication to systemic therapy or non-tolerance to systemic chemotherapy underwent treatment with DEBIRI TACE after a tumour board review. All the patients had liver-limited disease. The presence of metastases that exceeded 70% of the liver volume and the occurrence of metastases outside the liver were considered exclusion criteria. All patients had a life expectancy longer than three months and an Eastern Cooperative Oncology Group (ECOG) score equal to 2 or lower before the first DEBIRI TACE treatment.

### Data analysis

All data were obtained by reviewing patient files. The following variables were collected – baseline demographic and clinical data (age, sex, ECOG performance status, tumour location), periprocedural complications, duration of hospital stay, previous cycles of systemic therapy, number of metastases, type of liver impairment (unilobar or bilobar), values of tumour markers carcinoembryonic antigen (CEA) and cancer antigen 19-9 (CA 19-9) before and after treatment, radiological tumour progression, and survival. Limit values for tumour markers were used based on estimated upper normal plasma levels (for CEA ≤ 5 µg/L, CA19-9 ≤ 37 kU/L). Variables assessed as possible prognostic factors were age, ECOG status, tumour location, previous systemic therapy, number of metastases, uni- or bilobar disease, CEA and CA 19-9 before the first treatment, and rise or fall of tumour markers after the first treatment.

All adverse events were graded according to the Cardiovascular and Interventional Radiological Society of Europe (CIRSE) classification system for complications.^15^ Tumour response was assessed using the Response Evaluation Criteria in Solid Tumours (RECIST) and modified RECIST (mRECIST) criteria.

Survival was calculated as the time from the first DEBIRI to death or to the end of follow-up (July 20, 2020). Survival analysis was performed by using the Kaplan-Meier method. Progression-free survival (PFS) was calculated from the date of the first DEBIRI to disease progression or death from any cause. Survival endpoints for each factor were estimated according to Kaplan-Meier analysis and compared with the log-rank test. The p-values are two-sided and considered statistically significant at ≤ 0.05. Data were analysed using the statistical software SPSS 25 for Windows (IBM Corp., NY, USA).

### Treatment

Premedication included intravenous hydration, opioid analgesic, corticosteroid, antiemetic, and antibiotic prophylaxis. Intraprocedural pain was managed by a continuous intravenous infusion containing morphine (20 mg) combined with the nonsteroidal anti-inflammatory agent ketorolac (20 mg), starting two hours before the procedure for 24 hours. The procedure was performed in an angiography suite in local anaesthesia through the femoral approach. First, preliminary diagnostic angiography was performed to evaluate hepatic arterial supply. Then, a microcatheter (Progreat, Terumo Europe N.V, Belgium) was introduced into the left or right hepatic artery, followed by 2−4 mL intraarterial application of 1% lidocaine and 2 ml solution of microparticles loaded with 100 mg of irinotecan, respectively. Over time, the size of microparticles has changed noticeably. Initially, DC beads (Boston Scientific, Marlborough, Massachusetts) ranging between 100 and 300 micrometres in size were used. Later, there was a move towards using smaller particles, such as DC beads M1 ranging from 75 to 100 micrometres, and Tandem 100 micrometre beads (Tandem, Boston Scientific, Marlborough, Massachusetts) in the following years. The procedure was considered successful if at least 50% of the planned dose (50 mg of irinotecan-loaded beads) was delivered. In the case of unilobar disease, two treatments were performed at four-week intervals, and in the case of bilobar disease, four treatments were performed at two-week intervals. The patient’s vital signs and femoral access site were monitored after the procedure.

## Results

Between September 2010 and March 2020, 30 patients with histologically confirmed colorectal adenocarcinoma with liver-only metastases (22 men and 8 women; mean age 66,8 ± 13,2) were included in the study. DEBIRI TACE was conducted as a first-line treatment in 43% of patients unsuitable for systemic therapy and as salvage therapy after systemic therapy in 57%. In the second group, 100% (17 patients) were treated with the first line, 71% (12 patients) additionally with the second line and 47% (8 patients) with third-line systemic therapy. Eighty two percent of patients on systemic chemotherapy were treated in combination with targeted therapies. Patient characteristics are shown in Table 1.

**TABLE 1.** Patient demographics and clinicopathological features

**Age in years**	
Median (range)	68 (34–85)
Sex	**n (%)**
Male	22 (73)
Female	8 (27)
Primary tumour	
Colon	16 (53)
Rectum	14 (47)
ECOG performance status	
0	18 (60)
1	9 (30)
2	3 (10)
Liver metastases	
Unilobar	17 (57)
Bilobar	13 (43)
≤ 4 lesions	19 (63)
> 4 lesions	11 (37)
Previous chemotherapy	
Yes	17 (57)
No	13 (43)

### Treatment compliance and safety

113 DEBIRI procedures were performed with a median of 4 treatments per patient (ranging from 2 to 8). All procedures were technically successful. After the procedure, patients were hospitalised for a median of 4 days (ranging from 2 to 10 days). There were no treatment-related deaths or grade 4 and 5 adverse events. Non-serious adverse events were present in 53% of patients. Most of them were minor (grades 1 and 2). They contributed to post-embolic syndrome (PES) with significant abdominal pain in 43% of patients, vomiting in 6% of patients, nausea in 16%, diarrhoea in 3%, acute hypertension in 10%, and fever in 6% of patients. The PES symptoms were managed conservatively with hydration and non-steroidal anti-inflammatory drugs. The majority resolved in 48 hours. Seven (6%) high-grade adverse events (grade 3) occurred, including longer stay for pain management (n = 2), prolongation of hospitalisation due to the management of PES (n = 4) and PES requiring readmission (n = 1).

### Survival and prognostic factors

**FIGURE 1.**
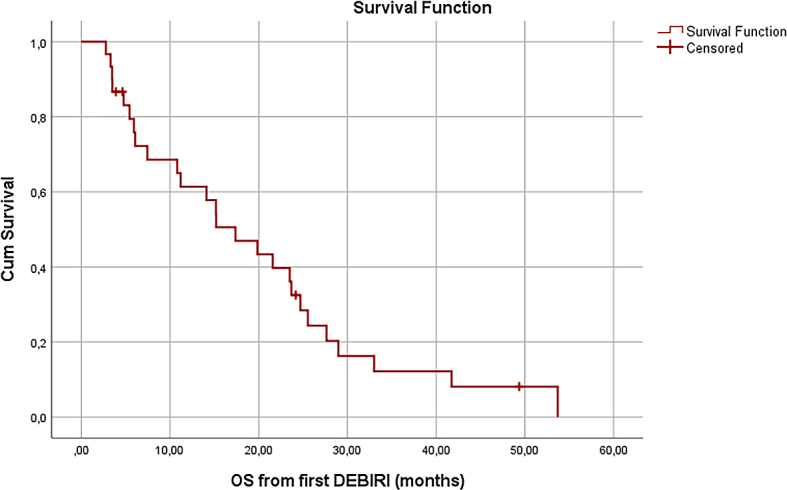
Overall survival (OS) from the beginning of irinotecan-loaded drug-eluting beads (DEBIRI) treatment.

**FIGURE 2.**
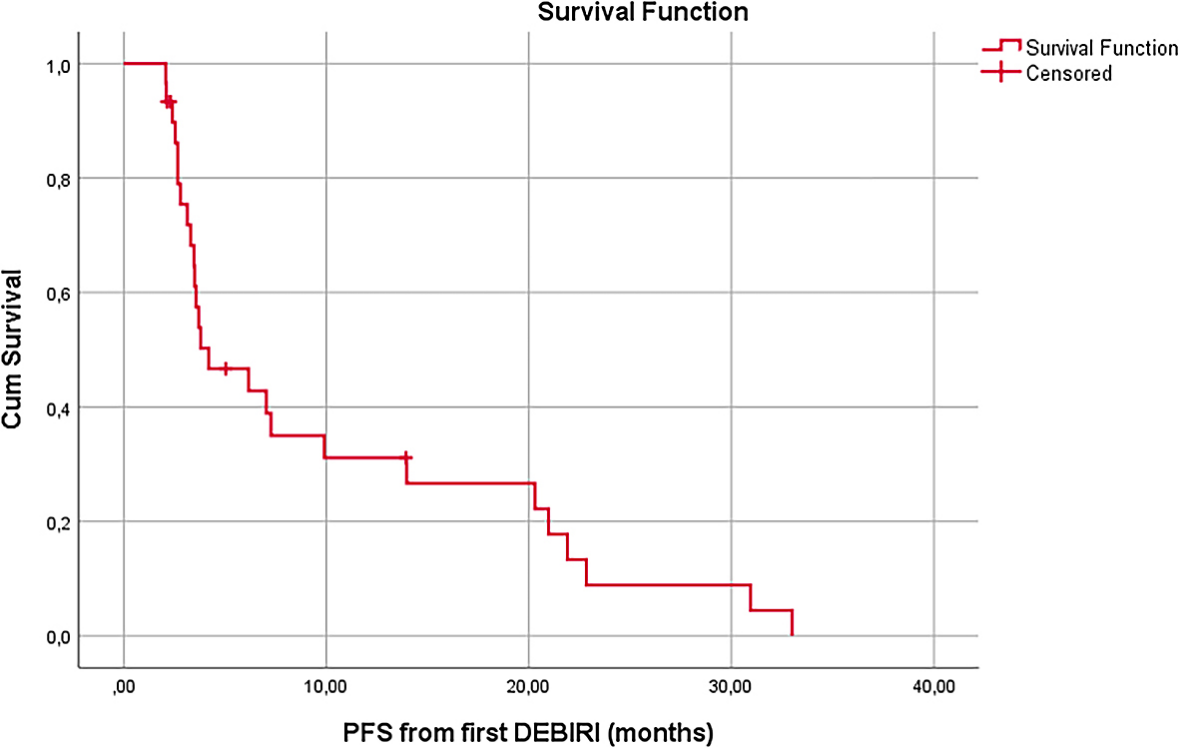
Progression-free survival (PFS) from the beginning of irinotecan-loaded drug-eluting beads (DEBIRI) treatment.

During the follow-up time, 26 of the patients died, and 4 remained alive. The median OS from the first DEBIRI TACE procedure was 17.4 months (95% confidence interval [CI]: 10,0–24,7 months) (Figure 1). The 1-year survival rate from the first DEBIRI TACE procedure was 61%, and 2-year survival rate was 25%. The median PFS from the first DEBIRI TACE was 4.2 months (95% CI: 0,9–7.4 months) (Figure 2). The most common site of progression was the liver (20 patients), with the lungs being the second (6 patients). Other progression sites included adrenal glands, lymph nodes, the primary tumour site and the vertebrae.

**FIGURE 3.**
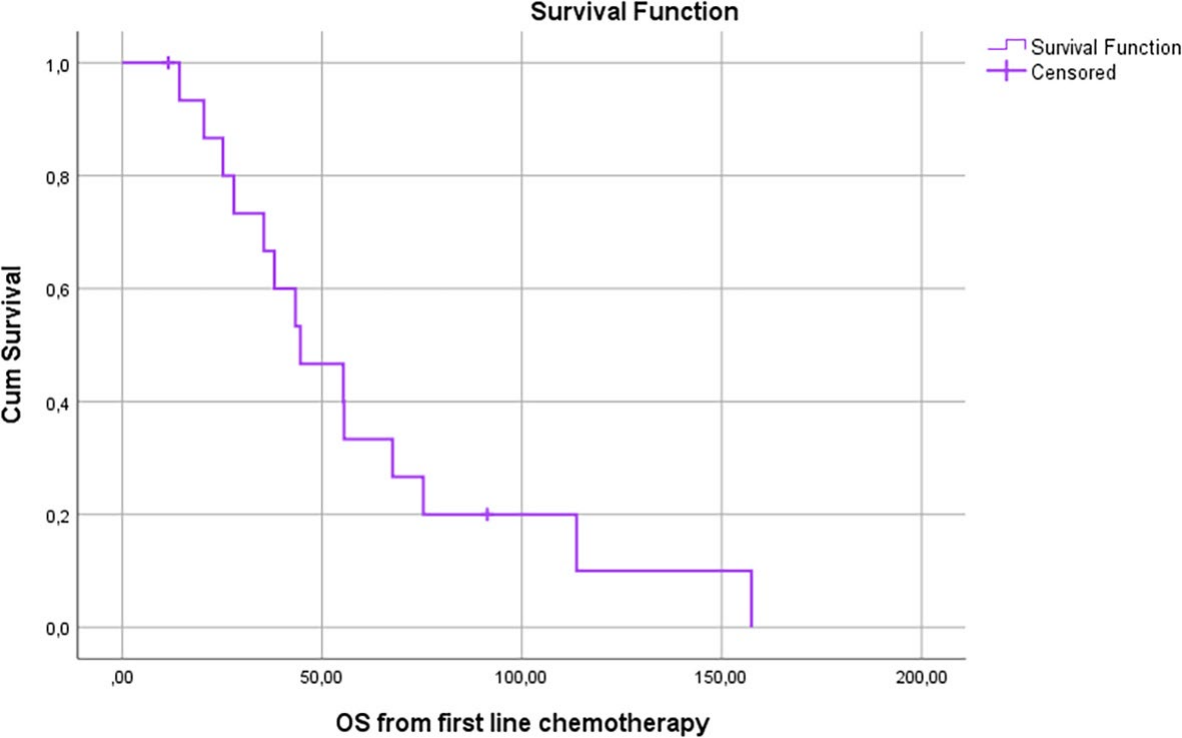
Overall survival (OS) of patients with prior systemic chemotherapy from the beginning of systemic treatment.

**FIGURE 4.**
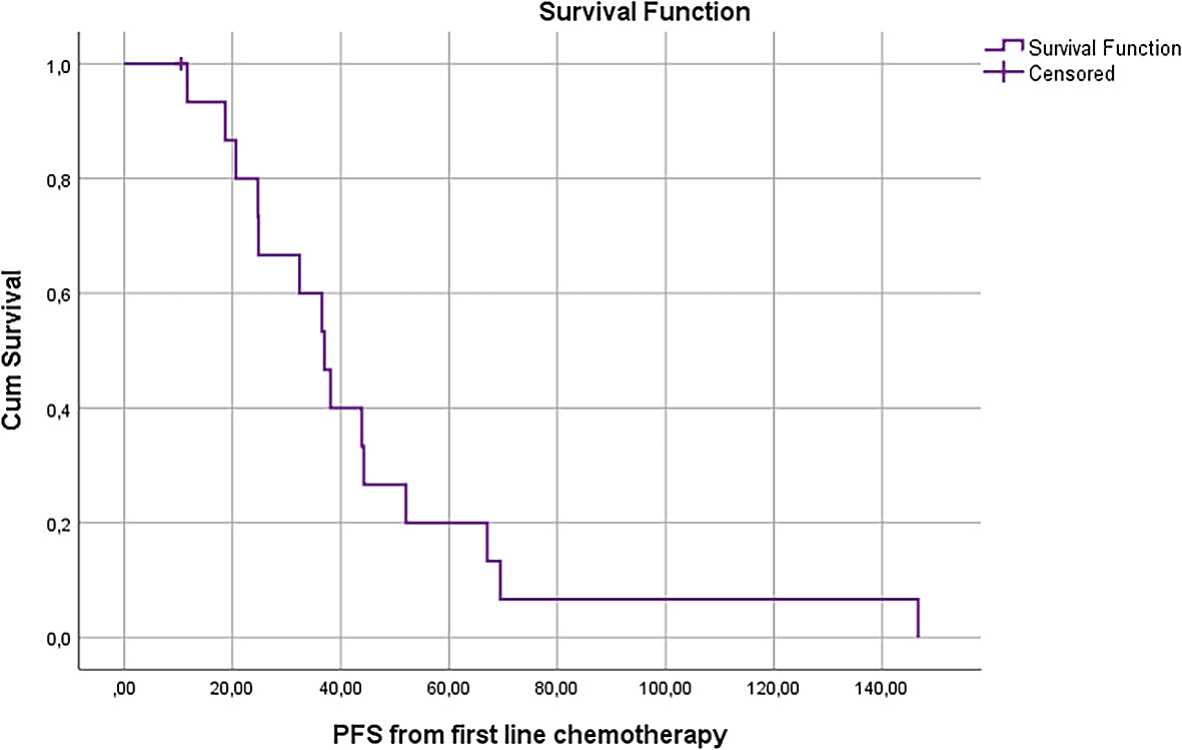
Progression-free survival (PFS) of patients with prior systemic chemotherapy from the beginning of systemic treatment.

In 17 patients treated with systemic therapy and DEBIRI TACE, median OS and PFS from the beginning of systemic therapy were 44.6 months and 37.0 months, respectively (Figures 3 and 4).

The median OS from the beginning of DEBIRI TACE in the 17 patients where DEBIRI TACE was used as salvage therapy was 17.4 months (95% CI: 11.0–23.7 months), and in the group without prior systemic therapy, the median OS was 21.6 months (95% CI: 3.8–39.4 months) (Table 2).

**TABLE 2.** Univariate analysis – influence of probable prognostic factors on overall survival

**Characteristics**	**n (%)**	**OS**	**95 % CI**	**p-value**
Age ≤ 65 years	11 (37)	15.2	8.5–21.8	0.284
Age > 65 years	19 (63)	21.6	4.7–38.4.	
Colon	16 (53)	23.7	16.8–30.5	0.145
Rectum	14 (47)	14.1	6.8–21.4	
ECOG 0	18 (60)	19.8	11.3–28.4	0.805
ECOG 1 or 2	12 (40)	17.4	10.0–29.7	
Previous chemotherapy	17 (57)	17.4	11.0–23.7	0.472
No previous chemotherapy	13 (43)	21.6	3.8–39.4	
Unilobar disease	17 (57)	23.5	7.4–39.6	0.106
Bilobar disease	13 (43)	15.2	1.9–28.4	
≤ 4 liver lesions	19 (63)	**23.5**	15.5–31.5	**0.002**
> 4 liver lesions	11 (37)	**10.8**	0.3–21.3	
CEA ≤ 5 µg/L before the first DEBIRI TACE	5 (27)	17.4	5.9–28.9	0.591
CEA > 5 µg/L before the first DEBIRI TACE	22 (73)	15.2	9.2–21.1	
Increase of serum CEA after first DEBIRI TACE	10 (33)	14.1	7.3–20.9	0.037
Decrease of serum CEA after first DEBIRI TACE	12 (40)	24.7	7.0–42.4	
CEA stayed the same	1 (3)	25.5		
CA 19-9 ≤ 37 kU/L before first DEBIRI TACE	16 (53)	15.2	10.1–20.2	0.393
CA 19-9 > 37 kU/L before first DEBIRI TACE	11 (47)	17.4	0.5–34.2	
Increase of serum CA 19-9 after first DEBIRI TACE	11 (48)	15.2	11.7–18.7	0.583
Decrease of serum CA 19-9 after first DEBIRI TACE	10 (43)	19.8	0.0–44.9	
CA 19-9 stayed the same	2 (9)	7.4		

CA 19-9 = cancer antigen 19-9; CEA = carcinoembryonic antigen; DEBIRI TACE = irinotecan-loaded drug-eluting beads transarterial chemoembolization; ECOG = Eastern Cooperative Oncology Group preformance status; OS = overall survival

Results from the univariate analysis between 10 clinical and radiological characteristics and OS are reported in Table 2. There were no data on levels of CEA and CA 19-9 for three patients before DEBIRI TACE treatment. Further, four patients had no data on CEA and CA 19-9 levels after the treatment. Univariate analysis showed better survival of patients with four or fewer liver metastases (p = 0.002). Age (p = 0.284), ECOG status (p = 0.805), tumour location (p = 0.145), previous systemic chemotherapy (p = 0.472), uni- or bilobar disease (p = 0.106), CEA and CA 19-9 before (p = 0.591;0.393) and after (p = 0.037;0.583) the treatment did not prove to be statistically significant predictors of survival.

## Discussion

The first-line treatment for patients with unresectable CRLM is chemotherapy with consideration of additional targeted therapies, usually anti-VEGF or anti-EGFR antibodies – a regimen usually well tolerated, even in elderly patients.^8,16^ When the liver is the sole or predominant site of metastases, and the response to systemic therapy is insufficient or systemic therapy is contraindicated or unsuitable, locoregional treatment options such as TACE should be considered.^7,8^ The introduction of DEBIRI TACE improved the ability to administer higher concentrations of irinotecan to liver metastases while reducing the systemic peaks of irinotecan, thus minimising side - effects. DEBIRI TACE has been proven safe and effective in treating CRLM and is more frequently used than in the past.^11,12,13^

In our study, DEBIRI TACE was conducted in 43% of patients as a first-line treatment and 57% as salvage therapy for patients who had received previous lines of systemic therapy (patients who did not tolerate more cycles of chemotherapy). Our study’s median OS from the beginning of DEBIRI TACE was 17.4 months, with progression-free survival of 4.2 months. This aligns with the previously reported trials with median OS and PFS for DEBIRI TACE of 18 months (ranging from 7.3 to 25) and 6.7 months (ranging from 4 to 11), respectively.^9,11^ In the salvage therapy group, the median OS was 44,6 months from the beginning of treatment with systemic therapy, confirming the usefulness of DEBIRI TACE as salvage therapy.

In our study, not all patients received systemic therapy before DEBIRI TACE treatment. Interestingly, the group without previous systemic therapy had longer OS from the start of DEBIRI TACE treatment than the previously treated group. Although the difference in survival is not statistically significant, it does raise a question as to whether TACE should be implemented sooner. One such study was done by Martin *et al*., comparing OS, PFS and tumour response between patients who underwent concurrent systemic therapy (*FOLFOX and bevacizumab*) and DEBIRI TACE and patients who were treated with systemic therapy alone. The group simultaneously treated with TACE had better tumour response in the first six months and longer PFS (15.3 months in the TACE arm versus 7.6 months in the arm with chemotherapy alone).^14^ These results require further exploration into the viability of DEBIRI TACE treatment not only as salvage therapy but also as consolidation treatment in combination with systemic therapy for unresectable CRLM. One of the potential reasons for better survival in patients without previous systemic therapy may be irinotecan resistance. Some authors report that DEBIRI TACE shows less efficacy if applied after previous systemic therapy due to irinotecan resistance.^11^ The reason for irinotecan resistance could be increased expression of EGFR receptors or active efflux, reducing the drug’s intracellular accumulation after the previously used chemotherapeutic agent irinotecan.^10,11,17^

Clinical and radiological factors that affect survival have yet to be determined. Our study is one of the first to ascertain prognostic factors affecting the survival of patients with CRLM treated with DEBIRI TACE. We found that patients with four or fewer liver metastases survived better than those with more. However, the size of lesions varies considerably; therefore, the number of lesions usually doesn’t give an accurate assessment of liver impairment. Thus, the metastatic volume would probably be a better predictive factor in further studies. One study where they used DEBIRI and capecitabine in heavily pre-treated patients found a statistically significant correlation between the decrease in CEA after the first DEBIRI treatment and survival.^18,19^ Another study found that patients with an ECOG of 0 had better survival than those with an ECOG of 1 or 2.^20^ While no such correlation was found in our research, more extensive studies should be performed to confirm these findings.

Safety of the procedure is also a primary concern, and we have shown that the number of significant adverse events (grade 3) is low, with only 6%. This is similar to recently published evidence on the CIREL registry, with 10% of grade 3 and 4% of grade 4 adverse events.^21^ The most common mild AE (grades 1 and 2) after the procedure is post-embolic syndrome (PES). The incidence of PES after DEBIRI TACE varies between studies, with a median incidence of 57%.^9^ Our study shows that 53% of patients had nonserious mild adverse events that contributed to PES, which is comparable to other studies.^9,21^ We assume that the low percentage of serious adverse effects is due to good premedication with antibiotics, antiemetic and intravenous hydration before and during the procedure and peri-procedural pain management with morphine and intra-arterial lidocaine.

DEBIRI TACE is a relatively new procedure in interventional oncology, performed primarily in larger centres on a specific group of patients; therefore, data from the literature are usually based on small populations. Such studies have difficulty recognising any essential prognostic factors that could affect survival, yet the lack of such clinical and radiological markers makes patient selection difficult.

The limitations of this study were retrospective design, the small number of patients evaluated, data on biomarkers and molecular targets not being collected, and the heterogeneity of the patient population.

In conclusion, DEBIRI TACE is a safe and effective treatment option for patients with CRLM refractory to systemic therapy. Our research found that four or fewer liver metastases correlated with better survival. Further studies are required to determine the role of DEBIRI TACE in treatment strategies for CRLM, as well as to recognise prognostic factors that would make patient selection easier.
